# Health and vulnerability to poverty in Ghana: evidence from the Ghana Living Standards Survey Round 5

**DOI:** 10.1186/2191-1991-2-11

**Published:** 2012-05-06

**Authors:** Jacob Novignon, Justice Nonvignon, Richard Mussa, Levison S Chiwaula

**Affiliations:** 1 Department of Economics, University of Ibadan, Ibadan, Nigeria; 2 Department of Health Policy, Planning & Management, School of Public Health, University of Ghana, Legon, Ghana; 3 Department of Economics, University of Malawi, Zomba, Malawi

**Keywords:** Poverty, Vulnerability to poverty, Health status, Welfare, Ghana

## Abstract

**Background:**

An understanding of the complex relationship between health status and welfare is crucial for critical policy interventions. However, the focus of most policies in developing regions has been on current welfare to the neglect of forward-looking welfare analysis. The absence of adequate research in the area of future poverty or vulnerability to poverty has also contributed to the focus on current welfare. The objectives of this study were to estimate vulnerability to poverty among households in Ghana and examine the relationship between health status and vulnerability to poverty.

**Method:**

The study used cross section data from the Fifth Round of the Ghana Living Standards Survey (GLSS 5) with a nationally representative sample of 8,687 households from all administrative regions in Ghana. A three-step Feasible Generalized Least Squares (FGLS) estimation procedure was employed to estimate vulnerability to poverty and to model the effect of health status on expected future consumption and variations in future consumption. Vulnerability to poverty estimates were also examined against various household characteristics.

**Results:**

Using an upper poverty line, the estimates of vulnerability show that about 56% of households in Ghana are vulnerable to poverty in the future and this is higher than the currently observed poverty level of about 29%. Households with ill members were vulnerable to poverty. Moreover, households with poor hygiene conditions were also vulnerable to future poverty. The vulnerability to poverty estimates were, however, sensitive to the poverty line used and varied with household characteristics.

**Conclusion:**

The results imply that policies directed towards poverty reduction need to take into account the vulnerability of households to future poverty. Also, hygienic conditions and health status of households need not be overlooked in poverty reduction strategies.

## Background

According to the World Health Organization (WHO), health is a “state of complete physical, mental and social wellbeing and not merely the absence of disease or infirmity” [1]. Health shocks constitute a sudden deterioration in the state of an individual’s health, caused by an illness and/or injury. The impact of health shocks on the welfare of individuals and households has been a major concern of policy makers. While shocks such as droughts and floods are seen to have significant effects on welfare, health shocks are unpredictable and pose a great challenge that any household has to face [2].

Somi et al [3] noted that health shocks and their associated costs have both short and long run impacts on household welfare. In the short-run, households that experience health shocks are forced to substitute consumption and production expenditure for health care. In the long-run, returns from investment in productive activities tend to reduce [3]. This implies that rational individuals will not only work towards improving their current health status but also their future state of health [4,5].Studies have shown that the occurrence of health shocks have significant negative impact on household current welfare [6-8]. In the case of Ghana, poor health has been shown to have devastating effects on economic growth and individual productivity [9,10]. An understanding of the complex relationship between health shocks and welfare is crucial for critical policy intervention.

Vulnerability to poverty is seen widely as a better future oriented/forward-looking measure of welfare [11-15]. The only study that analysed ex-ante welfare or vulnerability to poverty in Ghana did not consider the impact of health shocks [16].

Diseases such as malaria and acute respiratory infections continue to pose health problems in Ghana with high morbidity and mortality [17]. Further, about 29% of Ghanaians are estimated to be poor with the Greater Accra (11.8%) and Upper West (87.9%) regions being the least and most poor regions respectively (Table 1) [10]. The purpose of this study is to estimate a more forward-looking measure of welfare (vulnerability to poverty) and to examine how health shocks relate to and affect vulnerability to poverty in Ghana. Specifically, the study tests two null hypotheses; first, vulnerability to poverty does not vary with household characteristics. Second, household health status does not affect vulnerability to poverty. These are based on the intuition that household welfare depends on peculiar characteristics such as place of residence, gender of household head, etc. Also, poor health status is considered to reduce household wealth, hence making them more vulnerable to poverty [7].

**Table 1:** Summary of poverty incidence in Ghana

	**1991/92**	**1998/99**	**2005/06**
National poverty	51.7	39.5	28.5
Rural	64	50	39
Urban (Accra)	23	4	11
**Administrative regions**			
Western	59.6	27.3	18.4
Central	44.3	48.4	19.9
Greater Accra	25.8	5.2	11.8
Volta	57.0	37.7	31.4
Eastern	48.0	43.7	15.1
Ashanti	41.2	27.7	20.3
Brong Ahafo	65.0	35.8	29.5
Northern	63.4	69.2	52.3
Upper East	66.9	88.2	70.4
Upper West	88.4	83.9	87.9

Source: Ghana statistical service (2007).

### Poverty and vulnerability to poverty

In general, a household is considered to be poor if its current expenditure falls below a given threshold (poverty line). Specifically, the World Bank (WB) defines extreme poverty as living on less than US$1 per day and moderate poverty as less than US$2 or US$5 each day [18]. While most poverty studies have been faced with the challenge of identifying an appropriate welfare measure, household consumption expenditure or income is considered to be an acceptable measure of standard of living [19].

However, vulnerability to poverty is defined to include the probability that a household or an individual, whether currently poor or not, may be poor in the near future. For instance, Duflo [20] defined vulnerability as “a probability: the risk a household will fall into poverty at least once in the next few years”. This implies that, unlike poverty, vulnerability is a more future-oriented concept that considers possible changes in a household’s future welfare. While the above definition suggests that both the current poor and non-poor may be vulnerable to poverty, the degree of vulnerability depends on the risks faced by households and their ability to respond to these risks [21]. In Ghana, bush fires, infertile lands, snake bites and poor sanitary conditions have been identified as major risks faced by rural communities [22].

While various measures of vulnerability have been suggested in the literature, any measure of vulnerability should have two perspectives; the time horizon and the welfare measure. Hoddinott and Quisumbing [23] noted that the time horizon could vary, ranging from the next day to old age but consumption expenditure is mainly considered as the welfare measure. Three main concepts can be distinguished in measuring vulnerability to poverty; these are vulnerability as expected poverty (VEP), vulnerability as low expected utility (VEU) and vulnerability as uninsured exposure to risk (VER) [23,24].

The VEP approach defines vulnerability based on household expected consumption expenditure above a given threshold. While the VEU approach measures vulnerability as utility derived from some certainty-equivalent level of consumption and expected utility of consumption, the VER approach captures household welfare loss due to lack of effective risk management tools[23].

Defining vulnerability to poverty as the probability that a household’s consumption will fall below some benchmark (poverty line) in the near future, the current study employed the VEP approach to measure vulnerability. Two reasons motivated this choice; first, unlike VEU, VEP allows the use of cross section data in the estimation of vulnerability to poverty. Secondly, VEP estimates ex-ante welfare loss as opposed to VER which estimates ex-post welfare loss [23].

## Methodology

### Econometric Technique

Some studies on vulnerability have used panel data collected over a long period due to the forward-looking nature of the concept of vulnerability [11,25]. However, other studies have shown that cross section data can also be used in estimating vulnerability to poverty [12,14,16,26-29]. Following Chaudhuri [27], the probability of household *h*, finding itself to be consumption poor at time *t + j* can be expressed as;

(1) $$V_{\mathrm{ht}}=p_{r}(\ln C_{h,t+j}<\ln z)$$

Where $V_{\mathrm{ht}}$ represents vulnerability of household *h* at time *t*, $C_{h,t+j}$ is consumption of household h at time *t + j* and *z* shows poverty line of household consumption, *ln* is natural log.

The consumption generating process can be specified as;

(2) $$\ln C_{h}=X_{h}\beta +\epsilon_{h}$$

Where *C*_*h*_ is the per capita consumption expenditure for household *h*, *X*_*h*_ is observable household characteristics, *β* is a vector of parameters and $\epsilon_{h}$ is a zero-mean disturbance term that captures household’s idiosyncratic factors contributing to differential level of per capita consumption for households that share the same characteristics.

The use of cross section data makes it necessary for some assumptions to be made. First, the disturbance term, $\epsilon_{h}$ is log-normally distributed which implies that consumption expenditure, *C*_*h*_ is also log-normally distributed. Secondly, the structure of the economy is stable over time, ruling out the possibility of aggregate shocks (i.e. unanticipated structural changes in the economy). The first assumption is to enable the estimation of the probability that a household with characteristics, *X*_h,_ will be poor (household vulnerability level). The last assumption implies that uncertainties about future consumption stems solely from uncertainty about idiosyncratic shocks that the household will experience in the future. Vulnerability estimates should therefore be interpreted under the assumption that current economic structures will prevail, at least, in the near future. These assumptions may not be necessary in the case of sufficient and rich panel data that identifies the stochastic process of the economy’s structure.

Any given household *h*, with characteristics *X*_*h*_ can then have vulnerability to poverty level calculated using the estimated coefficients of equation (2) such that

(3) $$\hat{V_{h}}=p_{r}(\ln C_{h,t+1}<\ln z|X_{h})=\Phi \left(\frac{\ln z-X_{h}\hat{\beta }}{\hat{\sigma }}\right)$$

Where $\hat{V_{h}}$ is estimated vulnerability to poverty (i.e. the probability that per capita consumption level will be lower than the poverty line conditional on some household characteristics), Φ(.) is the cumulative density of the standard normal distribution and $\hat{\sigma }$ is the standard error from equation (2).

### Allowing for heteroscedasticity

Some studies that explore household consumption behaviour treat the disturbance term as stemming from measurement error and, thus, usually assume that the variance of the disturbance term is the same for all households. This assumption, as noted by Chaudhuri [14] leads to inefficient estimates not only in the main parameters of interest but also in the vulnerability estimates. This problem can be addressed by a simple functional form, which relates variance of the consumption function to household characteristics as follows:

(4) $$\sigma_{\epsilon ,h}^{2}=X_{h}\theta +\eta_{h}$$

A three-stage Feasible Generalized Least Squares (FGLS) procedure suggested by Amemiya [30] is used to estimate β and θ. Equation (2) is first estimated using the Ordinary Least Squares (OLS) procedure. The estimated residuals from equation (2) are then used to estimate the following equation by OLS

(5) $${\hat{\sigma }}^{2}{}_{ols,h}=X_{h}\hat{\theta }+{\hat{\eta }}_{h}$$

The predicted values from this auxiliary regression are used to transform equation (5).

(6) $$\frac{\sigma^{2}{}_{ols,h}}{X_{h}\hat{\theta }}=\frac{X_{h}}{X_{h}\hat{\theta }}\theta +\frac{\eta_{h}}{X_{h}\hat{\theta }}$$

Estimating equation (6) by OLS gives an asymptotically efficient FGLS estimate, ${\hat{\theta }}_{\mathrm{FGLS}}$. It can be shown that $X_{h}{\hat{\theta }}_{\mathrm{FGLS}}$is an efficient estimate of $\sigma_{e,h}^{2}$which is the variance of the idiosyncratic component of household consumption. Equation (2) is also transformed with the standard error of ${\hat{\theta }}_{\mathrm{FGLS}}$as follows;

(7) $${\hat{\sigma }}_{\epsilon ,h}=\sqrt{X_{h}{\hat{\theta }}_{\mathrm{FGLS}}}$$

(8) $$\frac{\ln C_{h}}{{\hat{\sigma }}_{\epsilon ,h}}=\left(\frac{X_{h}}{{\hat{\sigma }}_{\epsilon ,h}}\right)\beta +\frac{\epsilon_{h}}{{\hat{\sigma }}_{\epsilon ,h}}$$

OLS estimation of (8) yields an asymptotically efficient estimate of β. The estimated β_FGLS_ and θ_FGLS_ enable a direct estimation of expected log consumption (shown in equation 9) and expected variance of log consumption (shown in equation 10) respectively.

(9) $$E\left[\left(\ln{\hat{C}}_{h}|X_{h}\right)\right]=X_{h}\hat{\beta }$$

(10) $$Var\left[\left(\ln{\hat{C}}_{h}|X_{h}\right)\right]={\hat{\sigma }}_{h}^{2}=X_{h}\hat{\theta }$$

Finally, assuming that consumption is log normally distributed, vulnerability to poverty can be estimated as

(11) $${\hat{V}}_{h}=\Phi \left(\frac{\ln z-X_{h}{\hat{\beta }}_{\mathrm{FGLS}}}{\sqrt{X_{h}{\hat{\theta }}_{\mathrm{FGLS}}}}\right)$$

Thus, the estimation of vulnerability to poverty depends on such elements as the distributional assumption of normality of log consumption, the choice of poverty line, the expected level of log consumption and the expected variability of log consumption. The level of vulnerability to poverty reduces as expected consumption and expected consumption variability increases.

### Vulnerability to poverty threshold

The current study employed a vulnerability to poverty threshold of 0.5 as it is widely accepted as a reasonable threshold [26,31,32]. The following reasons justify the choice of the vulnerability threshold of 0.5. First, it makes intuitive sense to say that a household with a 50% probability of falling into poverty in the next period is vulnerable to poverty. Second, when a household - whose current level of consumption is equal to the poverty line - faces a zero mean shock; it has a one period ahead vulnerability of 0.5. In the limit, as the time horizon approaches zero then being currently poor and being vulnerable to poverty coincides [31]. Also Zhang and Wan [32] found that a vulnerability line of 0.5 provides a more improved prediction. This implies that households with estimated vulnerability to poverty above or equal to 0.5 are considered to be vulnerable to poverty.

### Poverty line

Two poverty lines were employed in the vulnerability estimations. These are an upper poverty line of GH¢370.89^1^Ghana Cedi to US Dollar exchange rate in 2006 (i.e. the data year) was GH¢0.917 = US$ 1.00 per adult per year and a lower poverty line of GH¢288.47 per adult per year to allow for sensitivity analysis [33]. The poverty lines were computed by the Ghana Statistical Service (GSS) from the Ghana Living Standards Survey (GLSS) and takes into account region-specific price differentials and nutritional needs of the population. Individuals whose consumption expenditure fell below the lower poverty line were considered to be extremely poor and were unable to meet their basic nutritional needs. On the other hand, individuals above the upper poverty line were considered as able to meet their nutritional requirements and purchase their basic non-food needs [33].

### Time horizon

This study argues that the certainty of the probability that a household or an individual will become poor exactly one period ahead is impractical and hence the time horizon should be any period in the future. The time horizon was therefore specified in this study as *t + j* instead of *t + 1*, where *j ≥ 1*[26,34].

### Data and variables

The study used 2005/2006 data reported in the Fifth Round of the Ghana Living Standards Survey (GLSS 5) conducted by the Ghana Statistical Service (GSS) with technical assistance from the WB and the European Union [35]. Nationally representative sample of 8,687 households in 580 enumeration areas (EAs), containing 37,128 household members were covered in GLSS 5 [10]. The EAs were first stratified into the 10 regions of the country and further into rural or urban place of residence. Ecological zones (i.e. Coastal, forest and northern savannah) of the country were also considered in the classification [36]. The GLSS 5 is the single largest country-wide household data available in Ghana and collects information on various aspects of household’s living conditions including education, health and employment. Furthermore, detailed information on household income and expenditure make the data very vital for a vulnerability study like this one. For the purpose of GLSS 5, a household is defined as a person or a group of persons, who live together in the same dwelling, share the same house-keeping arrangements and are catered for as one unit [10].

Household total food and non-food expenditure was used as the dependent variable in the expected consumption estimation. The variable includes a summation of all items purchased directly by household or indirectly (such as output from own production activities, batter exchange, transfer/remittances etc). Household health status was used as the main explanatory variable. The variable was proxied by standardizing the occurrence of illness and/or injury (i.e. the number of household members who had illness and/or injury during the two weeks preceding the survey divided by the total number of household members). This standardization presents a more representative measure of household health as far as the GLSS 5 in concerned. Admittedly, self-reported illness may contain some bias. However, this does not invalidate the results [37,38]. Household hygienic condition dummy was also included to complement household health status. The variable was measured by the presence or absence of safe drinking water and sanitary latrine in a household. Households without safe drinking water and sanitary latrines were considered to have unhygienic condition [12].

Other dependent variables include household size which is measured as the number of members in the household. Household head education dummies were included in four categories (no formal education, primary, secondary and tertiary education). Sex of household head was a dummy with a value of 1 if household head is male and 0 otherwise. Similarly, place of residence dummy was 1 if urban and 0 for rural residence. Age of the household head, measured in years, was also included in the model.

## Results

### Descriptive statistics

Table 2 shows that, on average, approximately three members per household reported illness and/or injury during the two week period that preceded the survey. Average annual household consumption expenditure on food and non-food items was GH¢1,190. Mean age of the household head was 45 years. Average household size was four (with a minimum of one and a maximum of 29).

**Table 2:** Summary of descriptive statistics

**Variable**	**Observations**	**Mean**	**Number (%)**
**Household characteristics**			
Number sick	8603	3.425317	
Good hygiene	8603		3392 (39.42)
Size	8603	4.281181	
**Head education**			
None	5609		1788 (31.88)
Primary	5609		2556 (45.57)
Secondary	5609		890 (15.87)
Tertiary	5609		375 (6.89)
Male head	8603		6202 (72.09)
Age of head	8603	45.35476	
Married head	8603		7783 (90.45)
Employed head	8603		7319 (85.07)
Urban residence	8603		5031 (58.48)
Good housing	8603		3847 (44.72)
Use communication			
facility			1121 (13.03)
Consumption expenditure (GH¢)	8603	1190	
**Administrative region**			
Western	8603		829 (9.24)
Central	8603		682 (7.93)
Greater Accra	8603		1226 (14.25)
Volta	8603		715 (8.310
Eastern	8603		901 (10.47)
Ashanti	8603		1561 (18.14)
Brong Ahafo	8603		793 (9.22)
Northern	8603		788 (9.16)
Upper East	8603		599 (6.96)
Upper West	8603		509 (5.12)
**Ecological Zone**			
Coastal	8603		2530 (29.41)
Forest	8603		3524 (40.96)
Savannah	8603		2549 (29.63)

Note: The exchange rate between the cedi and United States Dollar in 2006 was US$1.

### Vulnerability to poverty in Ghana

The average estimated vulnerability to poverty in Ghana was 56% (Table 3). This estimate reduced to about 49% when a lower poverty line was used (Table 4). While the Eastern region was found to have the highest average vulnerability of approximately 73%, the Upper West region had the least vulnerability with about 21% of the population being vulnerable to poverty. Other regions with relatively high incidence of vulnerability to poverty include the Western region (70%) and the Volta region (69%) (Table 3). Vulnerability to poverty was estimated to be 61% among urban households and 25% among rural households. Regarding gender, male-headed households were more vulnerable to poverty than female-headed households with mean vulnerability estimates of 58% and 51% respectively. Further, households located in the forest zones were more vulnerable to poverty with mean vulnerability of approximately 68%, followed by households in the coastal zones with mean vulnerability estimate of about 63%. Households in the savannah zones had the lowest average vulnerability of approximately 33%.

**Table 3:** Vulnerability to poverty profile for various population characteristics

	**Population share**	**Mean vulnerability**	**Vulnerability to population ratio**
Total	100	56	100
**Administrative region**			
Western	9.64	69.96	12.04
Central	7.93	64.37	9.11
Greater Accra	14.25	59.79	15.21
Volta	8.31	68.53	10.16
Eastern	10.47	73.47	13.73
Ashanti	18.14	61.63	19.96
Brong Ahafo	9.22	60.15	9.91
Northern	9.16	24.87	4.07
Upper East	6.96	28.38	3.54
Upper West	5.92	21.41	2.27
**Residence**			
Urban	41.52	61.06	45.27
Rural	58.48	52.42	54.75
**Gender of household head**			
Male	72.09	57.88	74.52
Female	27.91	51.15	25.00
**Ecological zones**			
Coastal	29.41	63.32	33.25
Forest	40.96	67.62	49.46
Savannah	29.63	32.68	17.29

Note: Upper poverty line of GH¢370.89 was used for the profiles above.

**Table 4:** Vulnerability to poverty profile for various population characteristics

	**Population share**	**Mean vulnerability**	**Vulnerability to population ratio**
Total	100	49	100
**Region**			
Western	9.64	63	12.39
Central	7.93	57	9.22
Greater Accra	14.25	43	12.51
Volta	8.31	66	11.18
Eastern	10.47	69	14.73
Ashanti	18.14	50	18.51
Brong Ahafo	9.22	57	10.73
Northern	9.16	24	4.49
Upper East	6.96	28	3.98
Upper West	5.92	21	2.53
**Location**			
Urban	41.52	47	39.82
Rural	58.48	51	60.86
**Gender of household head**			
Male	72.09	51	75.04
Female	27.91	44	25.06
Coastal	29.41	51.46	30.88
Forest	40.96	60.7	50.73
Savannah	29.63	31.97	19.33

Note: Lower poverty line of GH¢288.47 was used for the profiles above.

Results from the chi-square test of independence (Table 5) shows that there is no association between poverty and vulnerability to poverty (P value = 0.177).

**Table 5:** The vulnerable and the poor (percent)

	**Vulnerable**	**Non-vulnerable**	**Total**
Poor	55.69	44.31	100
Non-Poor	57.64	42.36	100
Total	56	44	100
Pearson Chi2 (1): 1.8233			
Probability: 0.177			

Note: Upper poverty line of GH¢370.89 was used.

### Determinants of vulnerability to poverty

Vulnerability to poverty was found to be lower for households with less number of ill members and this was significant at 1%. Also, the household hygienic condition dummy variable significantly (1%) relates to lower expected mean of consumption (Table 6). Further, households with large family sizes were found to be more vulnerable to poverty. This relationship was significant at 1%. Furthermore, household heads with higher formal educational attainments were less likely to be vulnerable to poverty relative to those with no formal education. The relationship was significant at 1% for household heads with tertiary, secondary and primary education attainments. Relative to unmarried household heads, married heads were less vulnerable to poverty with higher average future consumption. The relationship was significant at 1% level.

**Table 6:** Determinants of vulnerability to poverty

**Variables**	**Ex-ante mean consumption**	**Ex-ante variance consumption**
**Household Characteristics**		
Health status	−0.30639***	0.01033
	−0.03609	−0.09918
Good hygiene	0.16183***	0.08401
	−0.02351	−0.06515
Size	−0.12316***	−0.02088
	−0.00506	−0.01415
Head age	0.02091***	0.01127
	−0.00545	−0.01473
Head age square	−0.00021***	−0.00006
	−0.00006	−0.00016
Head married	−0.19835***	0.01917
	−0.03901	−0.10888
**Head Education**		
Primary	0.22626***	−0.14390**
	−0.02618	−0.07265
Secondary	0.51450***	−0.06216
	−0.03693	−0.09956
Tertiary and above	1.06162***	0.00089
	−0.05354	−0.13829
Head employed	0.14239***	−0.08746
	−0.03869	−0.10081
Urban residence	0.29912***	0.09134
	−0.02869	−0.0797
Good housing	0.22301***	0.01464
	−0.0277	−0.07748
Use communication facility	0.15592***	0.0459
**Administrative region**		
Central	−0.02749	−0.07701
	−0.04932	−0.14131
Greater Accra	0.06934	0.13945
	−0.04903	−0.13309
Volta	−0.33862***	0.06
	−0.04983	−0.13899
Eastern	−0.17312***	0.05841
	−0.04624	−0.12968
Ashanti	0.14176***	−0.02536
	−0.04295	−0.12256
Brong Ahafo	−0.20193***	0.10328
	−0.05456	−0.15242
Northern	−0.53127***	0.19594
	−0.08192	−0.2344
Upper East	−0.60534***	0.04238
	−0.08198	−0.24385
Upper West	−0.81322***	1.05060***
	−0.13048	−0.27623
**Ecological zone**		
Coastal	−0.00858	0.15757
	−0.05711	−0.16561
Forest	0.00564	0.16839
	−0.04839	−0.14117
	−0.03242	−0.08696
Constant	14.12813***	−2.31089***
	−0.12112	−0.33598
No. of Observations	8603	8603
R-squared	0.4	0.008
Adjusted R-squared	0.4	0.004
F-value	151.89***	1.83***

Note: 1. The dependent variable for the first estimation is the ex-ante mean of consumption.

2. The dependent variable for the second estimation is ex-ante variance of consumption.

3. Values of standard errors are reported in parenthesis.

4. ***significant at 1%; **significant at 5%; *significant at 10%.

## Discussions

Table 3 shows that 56% of the Ghanaian population was vulnerable to poverty compared to the observed poverty level of about 29%. The estimate, however, reduced to about 49% when the lower poverty line was used (Table 4) [16]. This finding implies that even though current policy on poverty alleviation considers the observed 29%, it is important not to focus all attention on this observed figure to the neglect of the twice that number that may be vulnerable to poverty in future. Otherwise, poverty alleviation becomes a programme that would be needed at all times (i.e. now and in the future). Considering both the current poor and the future poor ensures that poverty is tackled from a broader perspective in current and future generations.

Interestingly, rural households had lower average vulnerability to poverty than urban households. This means that, though current rural poverty stands at 39% - higher than urban poverty of 11% - urban households are comparatively more vulnerable to (future) poverty. This result contradicts the findings of earlier studies that vulnerability is higher in rural areas than in urban areas [12,16]. One reason that could be speculated for this result is the increase in rural–urban migration. The GLSS report indicated that about four in every ten residents in urban areas were in-migrants [10]. Such in-migrants face an enormous challenge posed by the high standard of living and lack of jobs. Further, the finding supports the premise that poverty and vulnerability to poverty are not necessarily the same and need to be treated as such.

Again, vulnerability to poverty was found to be higher among male-headed households than in female-headed households [39]. This finding confirms the findings of Appiah-Kubi et al. [16] who reported a lower vulnerability estimate of 36.3% for female-headed households compared to male-headed households with estimated vulnerability of 54.4%. Current poverty estimates show that out of the ten regions in Ghana, Upper West Region and Eastern Region have the highest and second lowest poverty estimates (Table 1). However, this study found that with respect to vulnerability to (future) poverty, Eastern Region is most vulnerable to poverty (73.47%) whilst Upper West Region is least vulnerable to poverty (21.41). A possible reason for this finding is the role of health status (discussed below) in influencing vulnerability to poverty. In Ghana, Eastern Region has consistently reported the highest HIV prevalence (with 4.9% in 2006) whilst Upper West Region reports lower HIV prevalence - 2.5% in 2006 i.e. the second lowest prevalence in the country [40]. While this result may not be intuitively appealing, it confirms the hypothesis that the current poor are not necessarily the vulnerable.

The results of the study further show that the current health status of a household plays a crucial role in determining the household’s vulnerability to poverty; as the general household health declines, future consumption is expected to reduce, making the household vulnerable to poverty in future. A complement of this finding was seen in the household hygiene condition variable which implies that households with good hygienic conditions were more likely to have good health, hence improved welfare. This finding confirms that health is both a consumption and investment commodity as good health enables individuals to engage in productive activities that translate positively into their consumption and investment activities. Similar result was found by Azam and Imai [12] in Bangladesh. Thus, better health status is an important vulnerability-reducing variable which has to be taken into consideration in designing policy interventions. In Ghana, health workforce and infrastructure are still relatively inadequate and sanitation conditions are relatively poor [17]. Hence, efforts at improving access to basic health services are crucial as argued by Nonvignon and Aglobitse [41] and Nonvignon et al. [42]. Good hygiene practices also need to be promoted at the household level. These interventions will not only facilitate the achievement of the health-related Millennium Development Goals but also translate into improving current and future welfare by reducing future poverty.

The results further suggest that education and household size are significant determinants of vulnerability to poverty apart from health status. These results are expected as education and family size directly or indirectly influence household consumption and welfare. Thus, the inclusion of strategies that improve the education of households in policies directed towards reducing vulnerability to poverty is not out of place. Such policies may include the reduction in cost of education and the provision of improved facilities, while encouraging female education at higher levels in both rural and urban areas.

A limitation of the study was the lack of panel data with sufficient length and richness which would have provided inter-temporal consumption expenditure for household vulnerability assessments. That is, changes in household expenditure and shocks overtime are better capture in panel data that provide information on the same household over time. The study was also limited by its inability to control for the existence of a possible simultaneity problem due to the lack of a good instrument in the data. Simultaneity problem occurs when a dependent and independent variables influence each other at the same time.

In this study, this problem may exist in the sense that, while health status may affect vulnerability to poverty, a reverse relationship may also exist. While the effect of health on vulnerability may be overstated, in the presence of simultaneity, the focus of this study is to establish a causal relationship between the two variables.

Further, while broader concepts of poverty exist (e.g. household income, assets etc), this study employs household expenditure as a measure of poverty. Future research could consider the above mentioned limitations.

It must also be noted that, in addition to estimating vulnerability to poverty, a major contribution of the current study lies in estimating the relationship that exist between vulnerability and health status which has received very little attention, especially in developing countries. However, generalizing the findings of this study should be with caution as countries may have different situations of poverty and issues of sensitivity relating to poverty.

## Conclusions

The study sought to examine the effect of health status on vulnerability to poverty among households in Ghana. The study findings underscore the significance of future poverty and confirmed the notion that poverty and vulnerability to poverty are different concepts. The study also found that health status affects vulnerability to poverty as the occurrence of illnesses is likely to reduce household future consumption.

An important step in improving household welfare and enhancing development will, therefore, be to adopt integrated programmes for the education and health sector. This will go a long way to improve health status and human capital which are critical to the growth and development of any population.

## Endnotes

^a^Ghana Cedi to US Dollar exchange rate in 2006 (i.e. the data year) was GH¢0.917 = US$ 1.00.

## Competing interests

The Authors declare that they have no competing interests

## Authors’ contributions

JN (1) and JN (2) conceived the study. JN (1), LC and RM undertook the analysis. JN (1) wrote the manuscript and JN (2) and LC reviewed the manuscript. All authors read and approved the final manuscript.

## Footnotes

^1^Ghana Cedi to US Dollar exchange rate in 2006 (i.e. the data year) was GH¢0.917 = US$ 1.00
